# Nocturnal noise and habitat homogeneity limit species richness of owls in an urban environment

**DOI:** 10.1007/s11356-019-05063-8

**Published:** 2019-04-22

**Authors:** Arkadiusz Fröhlich, Michał Ciach

**Affiliations:** Department of Forest Biodiversity, Institute of Forest Ecology and Silviculture, Faculty of Forestry, University of Agriculture, al. 29 Listopada 46, 31-425 Kraków, Poland

**Keywords:** Urban ecology, Acoustic predators, Traffic noise, Habitat diversity, Habitat homogenization, *Strigiformes*

## Abstract

Habitat loss and fragmentation are listed among the most significant effects of urbanization, which is regarded as an important threat to wildlife. Owls are the top predators in most terrestrial habitats, and their presence is a reliable indicator of ecosystem quality and complexity. However, influence of urbanization on owl communities, anthropogenic noise in particular, has not been investigated so far. The aim of this study was to identify the role of noise and landcover heterogeneity in the species richness of owl assemblage in the urban ecosystem. Owls were surveyed in the city of Kraków (southern Poland) on 65 randomly selected sample plots (1 km^2^). The area of main landcover types, landcover diversity index, mean size of landcover patch, and nocturnal noise level were defined within the sample plots and correlated with owl species richness. Five owl species were recorded in the study area with forests as the dominant landcover type for Tawny and Ural owls, grasslands for Long-eared and Barn owls, and gardens for Little owls. In total, 52% of sample plots were occupied by at least one species (1–3 species per plot). The number of owl species was positively correlated with landcover diversity index and negatively correlated with nocturnal noise emission. This study demonstrates that species richness of owls in urban areas may be shaped by landcover heterogeneity and limited by noise intensity. This indicates that noise changes top predator assemblage, which in consequence may disturb predator-prey interactions within human-transformed habitats.

## Introduction

Rapid growth in human population and an increase in urbanization means that urban environments are becoming significant terrestrial ecosystems, which is anticipated to become the dominant ecosystem at the global scale (Melchiorri et al. 2018). Urbanization is regarded as a one of the main threats to wildlife due to the impact on natural ecosystems, which includes habitat loss, fragmentation, and homogenization (McKinney and Lockwood 1999; Mcdonald et al. 2008). Urbanization also leads to an increase of noise intensity (Nemeth et al. 2013; Proppe et al. 2013), artificial lighting at night (Molenaar and Sanders 2006), air pollution (Herrera-Dueñas et al. 2014), roadkills (Coffin 2007), and disturbance by human or domestic predators (Marzluff et al. 2001), which significantly alter ecological processes within urbanized areas and their surroundings.

One of the most striking effects of urbanization is anthropogenic noise, which disrupts acoustic signals used by many groups of animals. Noise could decrease the efficacy of communication (Leonard and Horn 2012; Nemeth et al. 2013; Shannon et al. 2016) and therefore has negative effects on mating success (Gordon and Uetz 2012) and maintenance of territories (Lengagne and Slater 2002). Apart from limiting intra-specific communication, noise disrupts predator-prey interactions, which are largely dependent on acoustic signals used to locate both prey and predator. Noise could reduce hunting efficiency of listening predators (Francis et al. 2012b; Mason et al. 2016; Senzaki et al. 2016; Agha et al. 2017) and impair the anti-predatory behaviors of listening prey (Shannon et al. 2016; Petrelli et al. 2017). This could force predators to change foraging techniques (Mason et al. 2016) and/or avoid habitats under high noise levels (Fröhlich and Ciach 2017, 2018). Noise also leads to an increase in vigilance of potential prey (Shannon et al. 2016; Petrelli et al. 2017) and has an impact on prey mortality (Francis et al. 2012b). In consequence, noise could alter ecosystem functioning and services (Francis et al. 2012a).

Most owls are acoustic predators (Mikkola 1983); thus, noise limits their foraging efficiency (Mason et al. 2016; Senzaki et al. 2016) and may influence nest site selection of some species (Fröhlich and Ciach 2017). Development of road networks, which are considered as a main source of anthropogenic noise, could force owls to abandon seemingly suitable habitats (Hindmarch et al. 2012), which can lead to a decrease in their population density (Silva et al. 2012). However, when large patches of primary habitats are available in the landscape, the negative effect of noise could be limited due to the presence of less disturbed parts within (Fröhlich and Ciach 2017, 2018; Shonfield and Bayne 2017). Despite the high level of noise, reduced availability of habitats, numerous roadkills (Coffin 2007), and flushes related with human presence (Hathcock et al. 2010; Scobie et al. 2014; Cavalli et al. 2016), many owl species inhabit highly urbanized areas (Galeotti 1990; Rullman and Marzluff 2014). Due to the availability of suitable nesting sites (Klein et al. 2007) and high prey abundance (Dravecký and Obuch 2009; Hindmarch and Elliott 2014), some owls could start breeding period earlier and have higher clutch size and reproductive success in urban habitats compared to natural ones (Rebolo-Ifrán et al. 2017; Kettel et al. 2018).

At the species level, several studies have analyzed the relationship between urbanization and the occurrence of owls, pointing to a negative effect of noise on single-species occurrence (Galeotti 1990; Silva et al. 2012; Hindmarch et al. 2012; Fröhlich and Ciach 2017, 2018). Results from natural habitats suggest that industrial activity has minimal effect on owls’ assemblage (Shonfield and Bayne 2017). However, factors shaping the entire owl communities within urban areas have not been investigated so far. The aim of this study was to determine habitat parameters influencing owl communities in an urban ecosystem and to investigate the relationship between anthropogenic noise level and species richness. Due to the varied habitat preferences (Mikkola 1983) and differences in sensitivity to artificial sounds of particular species (Scobie et al. 2016; Shonfield and Bayne 2017), we expected that the number of owl species inhabiting urban areas will be a valid indicator of habitat diversity. However, at the same time, we suspected that nocturnal anthropogenic noise level will negatively affect some species and reduce total richness.

## Methods

### Study site

This study was carried out in the city of Kraków (Southern Poland; 50°05′ N, 19°55′ E) with an area of 327 km^2^ and a population density of 2,331 persons/km^2^ (GUS 2016). The habitat mosaic of Kraków contains forests (11% of city surface), parks (3%), private gardens (18%), grasslands (23%), arable lands (14%), and municipal greenery of publicly available built-up areas—squares, road verges, and playgrounds (26%) (for detailed description of the study area, see Fröhlich and Ciach 2017, 2018). The city is located within breeding ranges of five owl species (Barn owl *Tyto alba*, Little owl *Athene noctua*, Long-eared owl *Asio otus*, Tawny owl *Strix aluco*, and Ural owl *Strix uralensis*). There are three other species breeding in southern Poland (Pygmy owl *Glaucidium passerinum*, Tengmalm’s owl *Aegolius funereus*, and Eagle owl *Bubo bubo*); however, their breeding habitats are located out of the study area (Tomiałojć and Stawarczyk 2003).

### Sample plot selection and owl surveys

To evaluate owl assemblages in the city, 65 sample plots located in an urban matrix were surveyed using mapping method with playback stimulation. Plots were randomly selected by using Quantum GIS Software (QGIS 2013) from the grid of 1 km × 1 km squared plots (for details, see Fröhlich and Ciach 2017, 2018). The area of adopted grid cell exceeded the area of the core part of the territory of owls potentially occurring in the study region (Sunde and Bølstad 2004; Framis et al. 2011; Lövy and Riegert 2013). Two surveys within the breeding season of 2015, early (March 01–31) and late (April 01–30), were conducted on each of the sample plots. There were at least 2 weeks between the early and late surveys.

Since high noise intensity may limit detectability of birds, efficiency of playback and proper density of playback points were previously field-tested in intensive-noise conditions (see Fröhlich and Ciach 2018). Tests revealed that playback points separated with a distance of 300 m from each other correspond with the 150-m wide audibility range of playbacks and territorial calls of owls and ensure that the loudspeaker and the owl’s response could be recorded. In consequence, the regular network of 13 playback points was distributed within each plot to enhance the full coverage of the entire plot. At each of the stimulation points, standardized soundtracks of 1-min recordings of courtship and contact calls of Little owl, Barn owl, Long-eared owl, Tawny owl, and Ural owl (adopted census order) were played back with a 3-W loudspeaker with standard amplitude (~ 50 dB) for each plot and each playback point. Recordings of species were separated by 1-min breaks, and on the completion of the soundtrack, 2 min was given for the owls to react. Sample plots were controlled in random order, and survey of the entire sample plot (13 playback points) was carried during a single night.

Since weather conditions could also disrupt the ability of researcher to hear owls (Zuberogoitia et al. 2018), plots were surveyed only on rainless and windless nights (0–1 Beaufort’s scale). To check whether results of the surveys were not influenced by thermal and humidity conditions, we compared the air temperature and the air humidity between days during which at least one owl species was detected and during which no calling birds were revealed. Both the mean air temperature and the mean air humidity did not differ significantly between surveys during which owls were detected and surveys during which owls were not recorded (surveys conducted in March: *t* = 0.82 and *p* = 0.416 for temperature and *t* = 1.54 and *p* = 0.129 for humidity; surveys conducted in April: *t* = 0.17 and *p* = 0.868 for temperature and *t* = 0.69 and *p* = 0.493 for humidity).

**Fig. 1:**
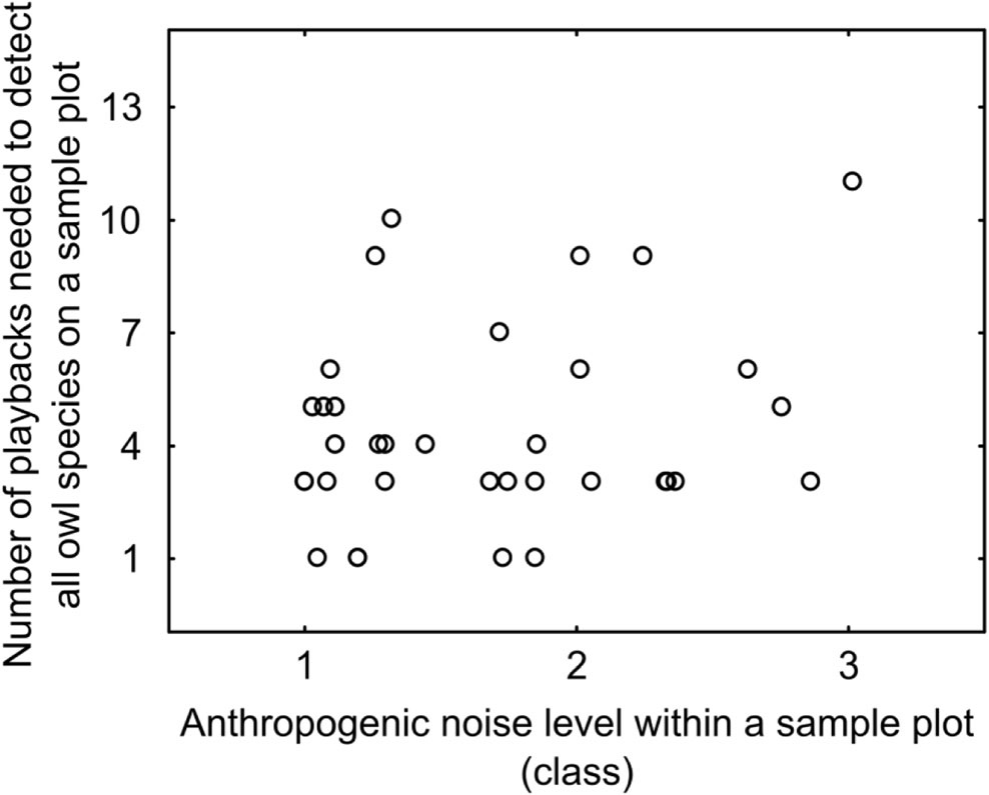
Relationship between the number of playbacks needed to detect all owl species within a sample plot (1 km^2^) and anthropogenic noise level; 13 is the total number of playback points surveyed within a sample plot

To minimize the effect of traffic noise on the audibility of playbacks and owl calls, surveys were conducted between 0:00 and 4:00 CET (conditions of lowest traffic noise level). To control the effect of noise on the detectability of birds, for each sample plot, we defined the number of playback points that enabled to detect all owl species recorded on a given plot. On average, all owl species recorded within a sample plot were detected on fourth playback point (median = 4, quartile range = 3–6, range = 1–11). Number of playbacks needed to detect all owl species within a sample plot was not correlated with anthropogenic noise level (Spearman’s rank correlation coefficient *r*_s_ = 0.16, *p* = 0.347; Fig. 1).

All nests, offspring, and calling adults (courtship and territorial calls) were plotted on the map backgrounded with satellite imagery (maps.google.com). To enhance the accuracy of detection, observers approached and/or moved around the alleged location of calling birds and located the exact tree/building with perching individual(s). Sample plots were defined as occupied by species if territorial behavior (courtship or territorial calls) or evidence of breeding (presence of nest or offspring) was recorded. The number of owl species recorded within each sample plot was used as a dependent variable.

### Landcover variables

The landcover variables within the boundaries of the sample plots were defined by using Quantum GIS Software (QGIS 2013) and polygon vector layers of the atlas of the real vegetation of Kraków (UMK 2012) and noise emission maps (MIIP 2016). Atlas is an effect of fieldwork done in 2006 and categorizes Kraków into 58 different landcover types. Polygons of the atlas were grouped into six main landcover types (forests, parks, grasslands, arable lands, gardens, and built-up areas), and vector layers were created for each of the types. The forest layer was made with polygons marked as a deciduous, coniferous, and mixed forests, and naturally growing shrubs. Parks and cemeteries, as commonly used by visitors, managed by the city authorities and containing wooded greenery were grouped into the parks layer. Gardens included areas covered by detached and semi-detached buildings mixed with large contiguous private green spaces such as backyard gardens, allotments, and orchards. Grasslands grouped meadows, pastures, uncultivated and fallow lands, swards, heaths, and the communities of trampled areas. Area of arable fields was used to create the arable land layer. Built-up area layer was made with polygons of terrains covered by compact and continuous buildings, blocks of flats, industrial infrastructure, and parking spaces mixed with small fraction of highly fragmented green spaces managed by the city authorities such as lawns, green squares, road verges, and playgrounds.

The proportion of each landcover type defined within plot boundaries was used to calculate the landcover diversity index (LANDCOVER_DIVERSITY) using the Shannon-Wiener formula. Because owls occurring on the study area represent two distinct habitat-dependent groups, forest specialists and open-habitat specialists, the area of two major landcover types was calculated within sample plots’ boundaries. Area of open landcover type (ha) was calculated by summing areas of grassland and arable lands (FARMLAND), and the area of forests and parks (WOODLAND) was summed to define area of wooded landcover type (ha). The layers of built-up areas representing publicly available greenery and gardens representing private greenery (along with buildings located within these layers) were used as a separate major landcover type (URBAN). Since all terrestrial environments were used for calculations of major landcover types, the total area of distinguished types was approaching 100% of the sample plot area (100 ha). Since some owl species are sensitive to habitat fragmentation (Redpath 1995), we also calculated the mean area of landcover patch (ha) within each sample plot (LANDCOVER_PATCH_AREA).

The nocturnal anthropogenic noise level variable (ANTHROPOGENIC_NOISE) was determined from the map of nighttime road noise emission (MIIP 2016). The map was based on measurements collected during a field campaign in 2012 on a regular net of sample points (100 m × 100 m) distributed within the city border (MIIP 2016). The map expressed in the form of a vector layer specified nine classes of noise intensity level (dB): (1) 40–44.9; (2) 45–50; (3) 50.1–55; (4) 55.1–60; (5) 60.1–65; (6) 65.1–70; (7) 70.1–75; (8) 75.1–80; and (9) 80.1–85 (MIIP 2016). Based on the map, for each sample plot, we calculated average noise class by using noise intensity classes weighted by the proportion of a given class within a given sample plot and use it as an explanatory variable. Accuracy of the anthropogenic noise emission map was previously confirmed with a field test (Fröhlich and Ciach 2018).

### Statistical analysis

To determine the environmental characteristics important for each species, percentage share of six main landcover types was calculated within sample plot. Median and quartile ranges for plots occupied by each owl species and for non-occupied plots were calculated. Principal component analysis (PCA) was performed to show relationship between distinguished major landcover types (FARMLAND, WOODLAND, and URBAN) and occurrence of each recorded owl species in form of dichotomous variables (species presence/absence). Spatial autocorrelation of the dependent variable was checked with Moran’s test (Rangel et al. 2010), which showed no spatial autocorrelation (for lag class 1 Moran’s *I* = 0.26, *p* = 0.257, for all next separation distances were close to zero and the semi-variance did not increase with lag distance). Generalized linear model with the Poisson log function (Bolker et al. 2009) was used to test the relationship between the number of owl species (species richness) and the area of wooded landcover type (WOODLAND), area of open landcover type (FARMLAND), landcover diversity index (LANDCOVER_DIVERSITY), the mean area of landcover patch (LANDCOVER_PATCH_AREA), and nocturnal anthropogenic noise level (ANTHROPOGENIC_NOISE). The starting model contains all variables, and the backward selection procedure was run until a model was obtained in which all of the variables analyzed were significant (*p* < 0.05). Since the area of URBAN landcover type was significantly correlated with other variables, this variable was excluded from modeling procedure. Prior to analyses, relationships between independent variables used in modeling procedure were tested with Spearman’s rank correlations (each of variable pairs correlated less than *r*_S_ = 0.5). The statistical procedures were performed using Statistica 12.0 software (StatSoft Inc. 2014).

## Results

**Fig. 2:**
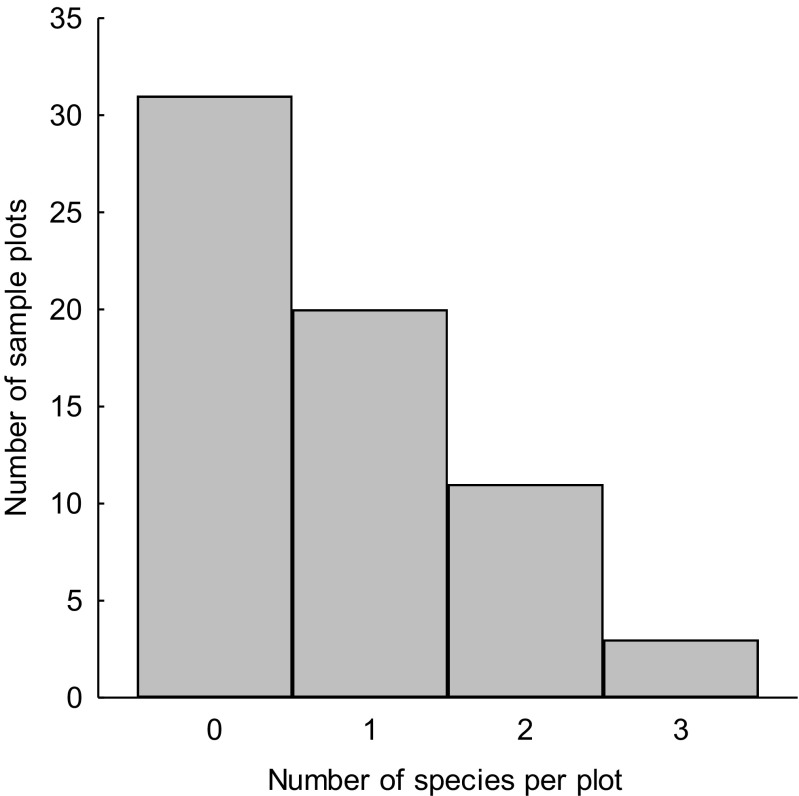
Number of owl species recorded on sample plots (1 km^2^, *N* = 65) located within an urban environment (Kraków, S Poland)

In total, five owl species were recorded on sample plots located within an urban environment (*N* = 65): Tawny owl (recorded on 40.0% of sample plots), Long-eared owl (18.5%), Little owl (13.8%), Barn owl (3.1%), and Ural owl (3.1%). 52.3% of sample plots were occupied by at least one owl species, and the number of species recorder per occupied plot varied between 1 and 3 (median = 1; quartile range, 1–2; Fig. 2).

**Fig. 3:**
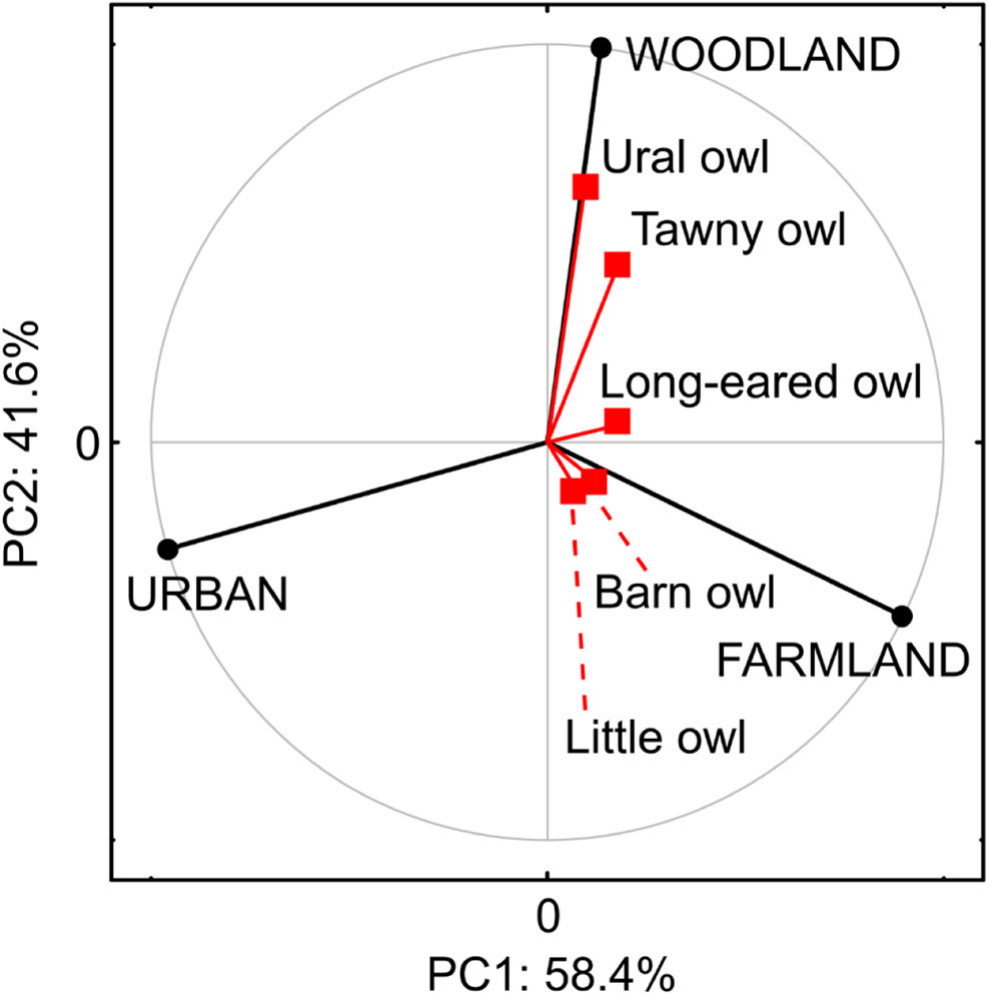
Results of principal component analysis (PCA) showing relationship between the presence of owl species (Barn owl *Tyto alba*, Little owl *Athene noctua*, Long-eared owl *Asio otus*, Tawny owl *Strix aluco*, and Ural owl *Strix uralensis*) and coverage of three major landcover classes (woodland, farmland, and urban) recorded on sample plots (1 km^2^) located within an urban environment (Kraków, S Poland)

Tawny owl and Ural owl occurrence demonstrated positive relationship with the area of wooded landcover type—forests and/or parks (Fig. 3; Table 1), while Long-eared owl, Little owl, and Barn owl tended to occupy plots dominated by farmland—grasslands and arable lands (Fig. 3; Table 1). The occurrence of all species showed negative relationship with the percentage share of urban landcover (Fig. 3).

**Table 1:** Median and quartile ranges of percentage share of main landcover types and class of nocturnal anthropogenic noise level on sample plots occupied by five species of owls and on non-occupied plots within an urban environment (Kraków, S Poland)

Species	*Strix aluco* (*N* = 26)	*Asio otus* (*N* = 12)	*Athene noctua* (*N* = 11)	*Tyto alba* (*N* = 2)	*Strix uralensis* (*N* = 2)	Non-occupied (*N* = 29)
FORESTS	19 (5–30)	9 (1–23)	5 (0–12)	21 (4–38)	49 (22–76)	2 (0–11)
PARKS	6 (0–16)	4 (0–12)	3 (0–11)	6 (3–8)	16 (16–16)	2 (0–5)
GRASSLANDS	15 (10–31)	20 (13–46)	12 (8–33)	30 (15–44)	6 (2–10)	25 (8–42)
ARABLE LANDS	1 (0–9)	7 (2–16)	3 (0–26)	6 (3–9)	1 (0–3)	0 (0–1)
GARDENS	18 (10–41)	17 (12–29)	18 (11–24)	26 (18–34)	8 (4–12)	18 (1–35)
BUILT-UP AREAS	3 (0–21)	8 (3–21)	5 (0–62)	8 (8–8)	16 (0–33)	12 (1–53)
ANTHROPOGENIC_NOISE	1.7 (1.1–2.0)	1.3 (1.1–1.9)	1.8 (1.1–2.7)	1.2 (1.1–1.3)	1.0 (1.0–1.0)	2.4 (1.9–2.7)

**Fig. 4:**
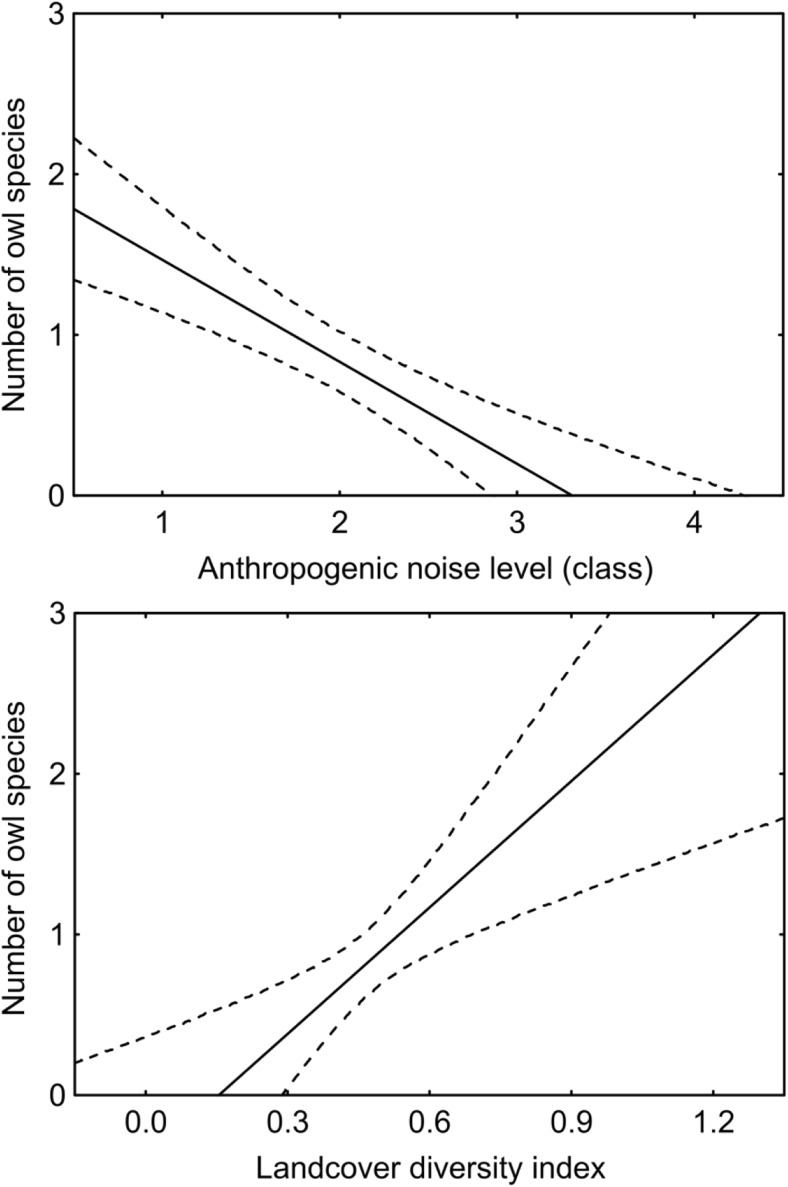
Relationship between the nocturnal anthropogenic noise level (top), the landcover diversity index (bottom), and the number of owl species recorded per sample plot (1 km^2^) located within urban environment (Kraków, S Poland)

The total number of owl species recorded in sample plots increased with increasing landcover diversity index (Table 2; Fig. 4), which was an apparent consequence of the varied habitat preferences of the particular owl species (Table 1; Fig. 3), but decreased with increasing level of anthropogenic noise level (Table 2; Fig. 4). The total area of wooded landcover type, the total area of open landcover type, and the mean area of landcover patch were not correlated with the total number of owl species (Table 2).

**Table 2:** Generalized linear model presenting the relationship between the number of owl species within an urban environment (Kraków, S Poland) and the area of woodland, farmland, landcover diversity index, mean area of landcover patch, and nocturnal anthropogenic noise level

Variable	Starting model					Final model				
	Estimate	SE	Wald’s stat.	95% CI	*p*	Estimate	SE	Wald’s stat.	95% CI	*p*
INTERCEPT	0.30	1.00	0.09	− 1.66 to 2.26	0.762	0.04	0.78	0.00	− 1.49 to 1.58	0.956
WOODLAND	0.01	0.01	2.34	0.00 to 0.03	0.126					
FARMLAND	0.00	0.01	0.00	− 0.02 to 0.02	0.982					
LANDCOVER_DIVERSITY	1.74	1.21	2.06	− 0.64 to 4.12	0.151	2.55	1.17	4.76	0.26 to 4.84	0.029
LANDCOVER_PATCH_AREA	− 0.02	0.02	0.66	− 0.06 to 0.02	0.418					
ANTHROPOGENIC_NOISE	− 0.86	0.26	10.71	− 1.38 to − 0.35	0.001	− 0.83	0.24	11.99	− 1.30 to − 0.36	0.001

## Discussion

Results of this study demonstrate that urban environments are settled by a relatively rich owl community, which uses various habitats of urban matrix, and the landcover diversity is positively correlated with species richness of the owls. This demonstrates that owl assemblage might be an indicator for landcover heterogeneity of the urban ecosystem and that landcover homogenization limits species richness. The heterogeneous habitat mosaic of the urban matrix creates a niche for several species, which vary in their habitat preferences (Mikkola 1983). It is worth emphasizing that all species ranging over the region (Tomiałojć and Stawarczyk 2003) were recorded within our study area. We found that species richness of owls is also limited by nocturnal anthropogenic noise pollution. Intensive noise has a limiting effect on the owl community since this group of birds is highly dependent on acoustic signals: owls use vocalization as a major mode of communication and most of owl species are typical acoustic predators. Populations of owls in noisy environments may achieve lower efficacy of hunting (Mason et al. 2016; Senzaki et al. 2016) and communication (Lengagne and Slater 2002), and distribution pattern of their territories is modified by noise pollution (Hindmarch et al. 2012; Fröhlich and Ciach 2017, 2018).

Our study is the first analysis of species richness of owls in an urban matrix. Studies conducted in non-urbanized habitats (homogenous oil palm plantation) revealed a positive relationship between the number of owl species and the presence of habitat patches increasing landscape heterogeneity (i.e., forests, houses, and trenches) (Yahya et al. 2016). Also, at a broader continental scale, habitat diversity was the main predictor of the number of owl species (Diniz-Filho et al. 2004). Habitat heterogeneity has a positive effect on species richness of owls due to the wide range of habitat preferences of different species (Mikkola 1983). Some owls depend on relatively homogenous habitats (mainly forest species), where they can find both nest sites and foraging areas (Mikkola 1983; Galeotti 1990; Fröhlich and Ciach 2018), while others are connected with farmland and require more complex habitat mosaics, which allow them to forage in open habitats (meadows and arable lands) and nest in woodlots and/or built-up areas (Mikkola 1983; Yahya et al. 2016; Fröhlich and Ciach 2017). Moreover, the differences in the availability and abundance of potential prey between habitat types of the urban matrix may shape habitat preferences of particular owl species. The increasing diversity of landcover types could also promote high diversity of potential prey. In consequence, increased diversity of small vertebrates could favor owl species with broad food niche. Owl assemblage recorded in our study consisted of woodland species (Tawny owl and Ural owl), species of the woodland-farmland mosaic and forest steppes (Long-eared owl), and typical farmland species (Little owl and Barn owl). The heterogeneous mosaic of urban matrix could therefore fulfill the habitat requirements of rich assemblage of owls.

Results of our study show that anthropogenic noise pollution has a negative impact on species richness of the owl community. A negative relationship between anthropogenic noise intensity and species assemblage is a novel finding for communities of acoustic predators. Negative effects of noise were shown for species richness of songbirds (Proppe et al. 2013), which due to lower efficacy of vocal communication have higher energetic expenditures for the establishment of their territories in noisy environments (Nemeth et al. 2013). As songbirds, owls use vocalization to advertise their territories to mates and to protect against rivals (Lengagne and Slater 2002). Moreover, begging calls of young and courtship calls of mates are crucial in sustaining family bonds in nightlight conditions (Mikkola 1983; Leonard and Horn 2012). However, some owl species may ignore specific type of noise (e.g., industrial; see Shonfield and Bayne 2017), what could be related to different frequencies of a particular type of noise. Owls use relatively high sound frequencies when foraging (Dyson et al. 1998); therefore, a noise characteristic is another factor which could potentially influence owls’ ecology, including their distribution. In our study, we used data on noise intensity (broadband average) since information on noise frequency is not available for spatial scales of our study area. Moreover, different species of owl may vary in their dependence on auditory cues while hunting (see Mikkola 1983; Dyson et al. 1998) and, therefore, the reaction to noise pollution could be species-specific. Further studies on the influence of noise characteristics, such as noise frequency and its timespan (i.e., duration and distribution of time with intensive noise), on ecology of owls are needed.

Earlier studies indicate that diurnal and nocturnal raptors’ species richness is consistent across the urbanization gradient (Bosakowski and Smith 1997; Rullman and Marzluff 2014). However, the reaction of raptor assemblage to urbanization could be species-dependent (Bosakowski and Smith 1997). Owls could show negative reaction not to urbanization in general sense but to certain aspects of that process. As demonstrated in our study, anthropogenic noise pollution could be an important factor. Other studies have revealed that other limiting factors include roadkill (Coffin 2007) and stress induced by humans or their pets (Hathcock et al. 2010; Scobie et al. 2014; Cavalli et al. 2016). However, despite the limiting effect of urbanization, owls find the cities to be suitable habitats. Birds born in urban environments may occupy these habitats again, showing a tolerance toward disturbance (Donázar et al. 2016). Previous studies have shown that owls benefit in urban areas due to a high abundance of prey, which are synanthropic rodents and birds (Dravecký and Obuch 2009; Hindmarch and Elliott 2014). The availability and high abundance of prey during the winter and early spring lead to an earlier breeding season for owls in urbanized habitats (Solonen 2013; Kettel et al. 2018) and higher reproductive success (Rebolo-Ifrán et al. 2017). Thus, the effect of urbanization on owls remains unclear and depends on the spatial context of habitat suitability, prey availability, and variation in noise pollution.

The results of our study show that urban environments are settled by a relatively high number of owl species compared to natural habitats (see Ciach and Czyżowicz 2014) and that their assemblage, apart from landcover heterogeneity of the urban mosaic, is shaped by nighttime acoustic conditions. Owls are the top predators in a wide range of habitats; thus, changes in their community could initiate disturbances in predator-prey networks and in consequence may shape ecosystems at a broader scale. Our findings of negative effect of anthropogenic noise on owl communities could be possibly applied to other habitats where anthropogenic noise is a permanent element of the environment, including farmland and woodland crossed by the dense network of roads or highways. This study gives outlines for designing of the urban landscape to maintain high species richness of nocturnal predators, which include maintaining large patches of woodland and farmland. Finally, we highlight the need to keep the natural habitats within urban matrix and limit anthropogenic noise pollution to preserve the conservation value of urbanized environments.
